# A joint triple extraction method by entity role attribute recognition

**DOI:** 10.1038/s41598-023-29454-7

**Published:** 2023-02-08

**Authors:** Xin Jing, Xi Han, Bobo Li, Junjun Guo, Kun Li

**Affiliations:** School of Computer Science and Engineering, Xi’an Technological University, Xi’an, Shaanxi China

**Keywords:** Computer science, Information technology

## Abstract

In recent years, joint triple extraction methods have received extensive attention because they have significantly promoted the progress of information extraction and many related downstream tasks in the field of natural language processing. However, due to the inherent complexity of language such as relation overlap, joint extraction model still faces great challenges. Most of the existing models to solve the overlapping problem adopt the strategy of constructing complex semantic shared encoding features with all types of relations, which makes the model suffer from redundancy and poor inference interpretability in the prediction process. Therefore, we propose a new model for entity role attribute recognition based on triple holistic fusion features, which can extract triples (including overlapping triples) under a limited number of relationships, and its prediction process is simple and easy explain. We adopt the strategy of low-level feature separation and high-level concept fusion. First, we use the low-level token features to perform entity and relationship prediction in parallel, then use the residual connection with attention calculation to perform feature fusion on the candidate triples in the entity-relation matrix, and finally determine the existence of triple by identifying the entity role attributes. Experimental results show that the proposed model is very effective and achieves state-of-the-art performance on the public datasets.

## Introduction

Entity and Relation Extraction (ERE) aims to extract conceptual objects and their interrelations from natural language texts according to sentence semantics, and form triples similar to (Subject, Relation, Object). As a key upstream task for applications such as knowledge graph construction, intelligent question answering, and public opinion analysis, it has always occupied an important position in natural language processing. Recent studies have shown that deep learning-based joint extraction methods can significantly improve the performance of ERE by effectively integrating the interaction features between entities and relations and alleviating the error propagation problem. However, the presence of complex linguistic phenomena such as SEO (Single Entity Overlap) and EPO (Entity Pair Overlap) (shown in Table 1, from TPLinker^1^) significantly and substantially increases the design complexity of joint extraction models, thus causing the problem that model construction becomes difficult to interpret. For example, the widely adopted model of the token semantic enhancement approach^2^, where the tokens of each word are spliced with other features (e.g., all types of relations) to form a synthetic encoding vector, is not only difficult to understand its ideological roots, but even flawed. The errors of this idea are obvious. First, the prediction method based on all types of relations is obviously less efficient than the search and selection method when faced with a large number of relations. Second, not all tokens in a sentence are relevant to the ERE task, and invalid and redundant information will not only increase the computational burden, but even interfere with the prediction results. Furthermore, it takes span (multiple consecutive tokens) to represent a concept in most cases, and whether a single synthetic vector can correctly represent the semantics of an entity or relational concept needs to be evaluated and verified. Thus, it is particularly important to propose explainable models that can reflect the essential process of knowledge extraction, which can help to solve the above problems and truly discover the laws of knowledge triple formation. In view of this, we simplify the design of the joint extraction model in terms of two criteria, namely, following an interpretable inference process and reducing redundant predictions, and expect to achieve the current state-of-the-art extraction performance. Our novel model benefits from the following two important inspirations.

**Table 1:** Example of the Normal, SEO and EPO overlapping patterns.

	Sentences	Triplets
Normal	[The United States] President [Biden] have a meet with [Elon Reeve Musk], the CEO of [Tesla]	(The United States, *president*, Biden) (Elon Reeve Musk, *the CEO of,* Tesla)
SEO	[Stephen Chow] is a famous comedian in [China], who was born in [Hong Kong]	(Stephen Chow, *national*, China) (Stephen Chow, *born in*, Hong Kong)
EPO	[Jay Chou] is the composer and singer of [“Nocturne”]	(Jay Chou, *the composer of*, Nocturne) (Jay Chou, *the singer of*, Nocturne)

First, inspired by the good question^3^ of why simply sharing encoders is detrimental to the accuracy of both independent entity and relationship extraction. The reason for this is that the two subtasks require different features to predict entity and relation types, and sharing features without semantic correlation is counterproductive. A well-designed pipeline method is also able to beat the joint method and obtain the latest SOTA score. Through an in-depth observation of this phenomenon, we argue that the reason why humans can achieve fast and accurate results in understanding the knowledge triples contained in texts is that they can identify target objects (both entities and relations) using only shallow text features (e.g., relational trigger words) and can verify the correctness of candidate triples guided by local semantics. Luo^4^ validates the conclusion that models with feature separation strategies have better performance than models with feature fusion strategies in joint extraction tasks, which is a strong support for this observation.

Second, inspired by TPLinker^1^, entities and their roles (subject or object) can be predicted separately, while disordered entity pairs and their roles can be effectively identified under relational conditions. Extending this idea, we believe that the subject-object role relationship is the most basic relationship that the triple must contain, so the problem of assignment relation type between disordered entities can be transformed into the process of triple role recognition. That is, for two entities in a sentence, if the E_1_ entity is the subject of the E_2_ entity and vice versa, then there must be a relationship between (E_1_, E_2_) that makes the subject-object relationship established. On the contrary, if there is no subject-object role relationship between (E_1_, E_2_), then obviously the probability that (E_1_, R, E_2_) is a true triple is almost zero.

Based on the above considerations, this paper proposes a novel end-to-end joint extraction model in terms of the roles that entities can play under certain relations, while incorporating a logically sound human knowledge extraction process. That is, when reading a sentence, humans can first identify the objects of entities and relations by shallow attention to the text, then they can use the identified objects to pre-assemble triples according to the role attributes, and finally they can verify the correctness of candidate triples under the guidance of complete sentence semantics. The model construction is simple, reasonable and interpretable because it is fully compatible with the human cognitive process. Since it is not limited by the sequence annotation and text generation model framework, it can naturally solve the overlapping relation extraction problem. At the same time, because only a limited number of relations are used for prediction, the noise from irrelevant relations is reduced, and the performance of the model is significantly better than that of traversing the relation set. Results of experiments on two widely used datasets, NYT^5^ and WebNLG^6^, show that our model beats most models that use fusion of the underlying features first and then classification, achieving competitive performance. This paper contributes as follows:

- We propose a new idea of joint triple extraction based on role attribute recognition of entities. Since the holistic semantic features of the triple better capture the correlation between entity pairs and relationships, it simplifies the process of formulating complex encoding layer feature fusion, thus effectively solving the problem of difficult interpretation of model structure.
- Combining new ideas with a rational cognitive process, we present an end-to-end triple extraction framework model. The model can realize the extraction of overlapping relations.
- Extensive experiments on two public datasets demonstrate that our model achieves comparable performance compared to state-of-the-art baselines.

### Related work

Since deep learning methods can automatically and efficiently extract task-related features from sentences, deep learning-based entity-relation extraction models have been receiving extensive attention. The earliest deep learning extraction models used the pipeline approach^7–9^. As the name implies, the method treats entity and relation recognition as two separate tasks, and optimizes two independent objective functions respectively. Most studies have concluded that this method has a natural drawback^10^ because it is formally unable to effectively interact the information between entities and relations. The verification of this assertion came to the opposite conclusion^3^, proving that the Pipeline method can also achieve excellent performance. Their experimental results clarify two things: ① It is important to learn the different contextual representations of entities and relations. ② Better performance can be obtained only by fusing entity type information in the input layer of the relation extraction model, and the interaction between the underlying features of entities and relations is not necessary. Therefore, although the underlying lexical features are shared in this paper, a feature separation strategy is used internally for entity and relationship prediction separately.

Compared with the lack of research enthusiasm for the Pipeline method, scholars have recently proposed a large number of neural network joint extraction models, and have achieved great success. For example, Zheng^11^ proposed a tagging framework that converts the joint extraction task into a tagging problem, which can directly extract entities and their relations without identifying entities and relations separately. Giannis^12^ uses a CRF (Conditional Random Fields) layer to model the entity identification task and models the relationship extraction task as a multi-headed selection problem, which enables the potential identification of multiple relations per entity. Wu^13^ proposed a method for enriching entity information that handles the relational classification task by locating the target entity and transmitting the information using a pre-trained architecture and merging the corresponding encodings of the two entities. However, while these efforts have solved the Normal and SEO problems, they have not yet had time to consider the more complex EPO issues.

Recent academic attention has focused on the challenge of overlapping relation extraction^14^. The approaches to solve this problem fall into two broad categories, sequence-based tagging and text-based generation. Sequence-tagging models treat the extraction task as a tagging problem, requiring the design of unique tagging schemes^1,15–17^. Text generation approaches use popular encoder-decoder architectures to generate triples^18–20^, similar to machine translation approaches. However, these dominant views largely employ strategies for constructing low-level interaction features under all types of relations^16,21–23^, and thus all suffer from redundant predictions and difficulties in interpretation. Different from the above methods, the model proposed in this paper only identifies limited relations in sentences, and only fuses entity and relation semantic features in the prediction stage, thus reducing the complexity of feature fusion in the encoding stage.

In addition, the information interaction between entity pairs and between entities and relations has been a key solution idea for joint extraction models. Fei^24^ dynamically learn the interactions between entity spans and their relation edges through a graph attention model, which achieves effective fusion of implicit features between entity pairs. Zhang^25^ apply a local focusing mechanism to entity pairs and corresponding contexts to obtain richer feature representations from local contexts to complete the RE task. Zheng^26^ modify the vanilla Transformer encoder with a weighted relative position attention mechanism, which can flexibly capture the semantic feature between entities. Liu^27^ models the relational graphs between the entities through a dynamic aggregation graph convolution module and gradually produces the discriminative embedded features and a refined graph through the dynamic aggregation of nodes. The commonality of these methods is that they can only satisfy the information fusion between two elements in a triad, i.e., the feature interaction between entity pairs or between a single entity and a relationship. However, a triple is composed of three elements, each of which has its own semantics and characteristics, and the feature interaction between any two combinations must be the key information for triple extraction. Therefore, this paper attempts to reduce the complexity of feature interaction by replacing the combination with the whole and learning the representations that determine the establishment of the triple directly from the three constituent elements.

### Methods

**Figure 1:**
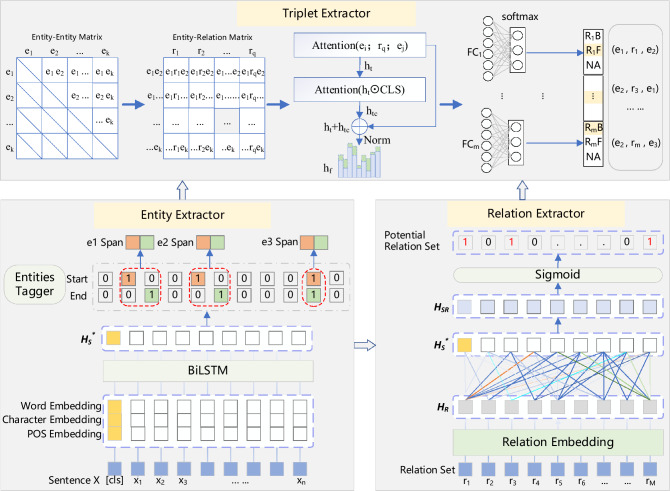
The framework of the proposed joint extraction model.

We believe that a correct triple, its three components must have their own implicit attributes and strong semantic correlation. The triple itself should contain characteristics such as relation pattern, subject role, and object role. According to this key idea, we design two mechanisms in the model framework. First, possible entities and relations are identified in parallel based on the underlying lexical features, and all possible candidate triples contained in the sentence are enumerated accordingly. This mechanism can reuse the entity pair information, and thus can easily perform overlapping relation identification. Secondly, the model uses residual connection and attention mechanisms to fuse triple features and perform role attribute label prediction based on them, thus ensuring fast extraction of valid triples. In the following, we will mainly introduce the novel framework model proposed in detail through three parts: entity extractor, relation extractor, and triple extractor based on role attributes recognition (as shown in Fig. 1).

#### Task definition

In the joint entity and relation extraction task, the goal is to identify all possible triples (Subject, Relation, Object) in a sentence. Towards this goal, we directly model the triples and design a training objective right at the triple level. This is in contrast to previous approaches^16,28^ that predict objects in the case of default subject roles. Formally, given an annotated sentence X=${\{{x}_{t}\}}_{t=1}^{n}$ and a set of triples T = {(S_e_, r, O_e_)} in X, our goal is to maximize the data likelihood of all sentences in the training set, which is defined as follows:

$$\mathop \prod \limits_{{\left( {S_{e} ,r ,O_{e} } \right) \in T}} p\left( {\left( {S_{e} ,r,O_{e} } \right){|}X} \right)$$

1 $$= \mathop \prod \limits_{r \in T} p\left( {r{|}X} \right)\mathop \prod \limits_{{\left( {S_{e} ,O_{e} } \right) \in T|r}} p\left( {\left( {S_{e} ,O_{e} } \right){|}X,r} \right)$$

$$= \mathop \prod \limits_{r \in T} p\left( {r{|}X} \right)\mathop \prod \limits_{{\left( {e_{i} ,e_{j} } \right) \in E}} p\left( {\left( {e_{i} ,e_{j} } \right){|}X} \right)\mathop \prod \limits_{{l_{O}^{S} \in L}} p\left( {l_{O}^{S} {|}X,r,e_{i} ,e_{j} } \right)$$

$$= \mathop \prod \limits_{r \in T} p\left( {r{|}X} \right)\mathop \prod \limits_{{e_{i} \in T}} p\left( {e_{i} {|}X} \right)\mathop \prod \limits_{{e_{j} \in T}} p\left( {e_{j} {|}X} \right)\mathop \prod \limits_{{l_{O}^{S} \in L}} p\left( {l_{O}^{S} {|}X,r,e_{i} ,e_{j} } \right)$$

where S_e_ = S→e and O_e_ = O→e represent entities that can act as subject and object. Here S is the subject role, O is the object role, e is the entity (Note that the formula adds a letter subscript to e in order to distinguish between two different arbitrary entities), and → is the property descriptor. That is, we split the usual sense of the S (subject) and O (object) symbols into two things, the entity and its role. r∈T represents the relation in the triple T. T|r is the set of triples with r as the relation in T. (S_e_, O_e_)∈T|r is an entity pair in T|r. E is the set of all entity pairs in x. Since the existence of two entities in an entity pair is an independent event (i.e. p(AB) = p(A)p(B)), the probability of the entity pair (e_i_, e_j_) can continue to be decomposed as the product of the probabilities of entities e_i_ and e_j_. L is the set of entity role labels under a relationship. $l_{O}^{S}$ is {R_m_F, R_m_B, Na}, representing the forward, reverse and disordered properties of the subject-object relationship of the entity pair, respectively.

#### Entity extractor

We use a multi-granularity representation vector concatenated by word embedding, character embedding and POS embedding as word embedding in a sentence. The word embedding is encoded by the pre-trained BERT^29^ that captures the overall contextual features of the words. The character embedding is generated by encoding using convolutional neural networks, which effectively capture morphological information about words. Similar to other methods^21^, we apply NLTK to tag sentences with POS, and randomize vector matrix of POS labels to get POS embedding. Through these methods, the embedding vector H_S_∈${\mathbb{R}}$ ^n∗d^ of a sentence can be obtained, where d_s_ = d_word_ + d_char_ + d_pos_, and d_word_, d_char_, d_pos_ represent the dimension of word embedding, character embedding and POS embedding respectively. To fully integrate multi-granularity information to improve entity and relationship recognition, BiLSTM layer is used to output the final sentence representation $H_{S}^{*}$. We use this underlying representation for both entity and relation recognition. The process of obtaining a sentence embedding representation is as follows:

2 $$H_{S} = \left[ {h_{1} ,h_{2} , \ldots ,h_{n} } \right] = BERT;Character;POS\left( {\left[ {x_{1} ,x_{2} , \ldots ,x_{n} } \right]} \right)$$

3 $$H_{S}^{*} = \left[ {h_{1}^{*} ,h_{2}^{*} , \ldots ,h_{n}^{*} } \right] = BiLSTM\left( {\left[ {h_{1} ,h_{2} , \ldots ,h_{n} } \right]} \right)$$

Our entity tagger does not distinguish between header and tail entities, but directly marks the start and end positions of all possible entities in the sentence. Specifically, we use two binary classifiers to classify the last layer output of the BiLSTM directly. The detailed operations of the entity tagger on each token are as follows:

4 $$\begin{array}{*{20}c} {p_{i}^{start} = \sigma \left( {W_{start} h_{i}^{*} + b_{start} } \right) } \\ \end{array}$$

5 $$\begin{array}{*{20}c} { p_{i}^{end} = \sigma \left( {W_{end} h_{i}^{*} + b_{end} } \right) } \\ \end{array}$$

where $p_{i}^{start}$ and $p_{i}^{end}$ represent the probability of identifying the *i*-th token in the input sequence as the start and end position of an entity, respectively. $W_{start}$, $W_{end}$, $b_{start}$, $b_{end}$ are learnable parameters. $\sigma ()$ is the sigmoid activation function. The corresponding token will be assigned with a tag 1 if the probability exceeds a certain threshold or with a tag 0 otherwise. We then use the proximity principle to match the start and end positions to form the entity span, and use [$h_{i}^{*} ;h_{j}^{*} ;width\left( {ij} \right)$] as entity span representation. Where *i* and *j* are the index numbers of the token, $h_{i}^{*}$ and $h_{j}^{*}$ are the feature representations of the span start and end token, respectively, and $width\left( {ij} \right)$ is a 20-dimensional embedding standing for the span width.

We define the training loss of entity extractor as the sum of the negative log probabilities of the true start and end tags by the predicted distributions:

6 $$\begin{array}{*{20}c} {L_{entity} = - \frac{1}{N}\mathop \sum \limits_{i = 1}^{N} \mathop \sum \limits_{{t \in \left\{ {start,end} \right\}}} \left( {y_{i}^{t} \log p_{i}^{t} + \left( {1 - y_{i}^{t} } \right){\text{log}}\left( {1 - p_{i}^{t} } \right)} \right)} \\ \end{array}$$

Here, N is the length of the given sentence. y_i_ represents the ground truth, and p_i_ represents the predicted value output by the *i*-th token of the sigmoid.

#### Relation extractor

This component, shown in the bottom right corner of Fig. 1, will output a subset containing the potential relations in the sentence. Unlike previous work^22,30^, the relation extractor employs the attention mechanism of the relation matrix on sentences. First, we use the method of RIFRE^16^ to generate relation embedding.

7 $$\begin{array}{*{20}c} {H_{R} = \left[ {R_{1} ,R_{2} , \ldots ,R_{m} } \right] = W_{r} E\left( {\left[ {r_{1} ,r_{2} , \ldots ,r_{m} } \right]} \right) + b_{r} } \\ \end{array}$$

where m is the number of predefined relations, r_i_ is the one-hot vectors of relation indices in the predefined relations, E is the relation embedding matrix, W_r_ and b_r_ is the trainable parameters, ${\text{R}}_{{\text{i}}} \in {\mathbb{R}}^{{d_{h} }}$ is obtained by mapping the vector r_i_ through a linear layer after embedding. The ${\text{H}}_{{\text{R}}} \in {\mathbb{R}}^{{m \times d_{h} }}$ relation embedding matrix is used in the following steps and is updated as the relation extractor is trained. Then, we need to calculate the similarity between H_R_ and $H_{S}^{*}$ to denote $H_{S}^{*}$ by H_R_. The attention-based potential relation embedding $H_{SR}$ can be obtained as:

8 $$Q = H_{R} W_{Q} , K = H_{S}^{*} W_{K} , V = H_{S}^{*} W_{V}$$

9 $$H_{SR} = Softmax\left( {\frac{{QK^{T} }}{{\sqrt {d_{k} } }}} \right)V$$

where ${\text{W}}_{{\text{Q}}} ,{\text{W}}_{{\text{K}}} ,{\text{W}}_{{\text{V}}} \in {\mathbb{R}}^{{d_{h} \times d_{k} }}$ are trainable parameters, $H_{SR} \in {\mathbb{R}}^{{m \times d_{k} }}$, d_k_ is dimension of Q、K and V.

10 $$\begin{array}{*{20}c} {P_{i}^{rel} = \sigma \left( {W_{rel} h_{{SR_{i} }} + b_{rel} } \right)} \\ \end{array}$$

Obviously, relation recognition can adopt the same multi-label classification method as entity recognition. As shown in Eq. (10), the relation tagger uses a sigmoid to obtain the probability of a potential relation after a linear feature extraction layer. If the probability exceeds a certain threshold, the corresponding token will be assigned as 1, otherwise it will be 0. Where $W_{rel}$, $b_{rel}$ are learnable parameters, and $\sigma ()$ is the sigmoid.

11 $$\begin{array}{*{20}c} {L_{relation} = - \frac{1}{M}\mathop \sum \limits_{i = 1}^{M} \left( {y_{i} \log p_{i}^{rel} + \left( {1 - y_{i} } \right){\text{log}}\left( {1 - p_{i}^{rel} } \right)} \right)} \\ \end{array}$$

We also used cross-entropy loss for the relation extractor. Where M is the number of predefined relations, y_i_ represents the ground truth, and $P_{i}^{rel}$ represents the predicted value output by the *i*-th token of the sigmoid.

#### Triple extractor based on role attributes recognition

The triple extractor performs entity-relation feature aggregation from a holistic perspective through residual connections, and then predicts the existence of a triple through entity role attributes. First, using the finite entities and relations predicted by the previous two modules, we can construct a finite number of candidate triples. As shown in the top left corner of Fig. 1, we enumerate all entity pairs using the entity-entity matrix. Due to the symmetry of the matrix, we only select out the set of data in the upper right corner of the matrix. Subsequently, using the entity-relation matrix we enumerate all the candidate triples. Assuming that a sentence contains K entities and Q relations, the number of candidate triples $\left| \varepsilon \right|$ can be calculated from the following equation. The minimum requirement for the presence of a candidate triple in a sentence is K ≥ 2 and Q ≥ 1.

12 $$\begin{array}{*{20}c} { \left| \varepsilon \right| = \frac{{K\left( {K - 1} \right)}}{2} \times Q} \\ \end{array}$$

Next, we use a residual concatenation module to obtain the triple fusion features. In the preliminary fusion layer, we regard triple as a simple graph composed of two entity nodes connected by a relation line, and fuse its features using the neighbor node information aggregation method of GAT (Graph Attention Networks)^31^.

13 $$\begin{array}{*{20}c} { h_{t} = f\left( {\left[ {W_{e} e_{i} ;W_{r} r_{q} ;W_{e} e_{j} } \right] + b_{t} } \right)} \\ \end{array}$$

where e_i_, e_j_ represent entity span representations, $r_{q} \in {\mathbb{R}}^{{d_{h} \times 1}}$ are relation vector, W_e_, W_r_, b_t_ are trainable parameters, and f represents a fully connected layer. Feature fusion in the second layer first uses dot product attention to extract valuable information from the global sentence semantic representation [CLS] and then sums it with the h_t_ vector. After that, the result followed by a layer normalization operation to prevent some values from being too large, and the output representation h_f_ is obtained.

14 $$\begin{array}{*{20}c} { h_{tc} = Softmax\left( {\frac{{h_{t} W_{tq} *\left( {CLSW_{tk} } \right)^{T} }}{{\sqrt {d_{t} } }}} \right)CLS} \\ \end{array}$$

15 $$\begin{array}{*{20}c} { h_{f} = LayerNorm\left( {h_{t} + h_{tc} } \right)} \\ \end{array}$$

where ${\text{W}}_{{{\text{tq}}}} ,{\text{ W}}_{{{\text{tk}}}} \in {\mathbb{R}}^{{d_{h} \times d_{t} }}$ are trainable parameters, ${\text{h}}_{{{\text{tc}}}} ,{\text{ h}}_{{\text{f}}} \in {\mathbb{R}}^{{1 \times d_{h} }}$. Finally, triple fusion features are input into relation-specific fully-connected networks for classification according to the relation type to determine whether there is a positive-order (Subject, Object), reverse-order (Object, Subject) or None role order attributes among the triple components, which can be formalized as:

16 $$\begin{array}{*{20}c} { p_{i}^{role} = softmax\left( {FC_{r} \left( {h_{f} } \right)} \right)} \\ \end{array}$$

where $p_{i}^{role}$ indicates the probabilities of role types such as R_m_F, R_m_B and Na (i.e., not a triple). If the predicted value is R_m_F, the result will be output in the order of the input of the candidate triples, and the opposite if it is R_m_B. NA can filter out low confidence triples.

17 $$\begin{array}{*{20}c} { L_{role} = - \frac{1}{M}\mathop \sum \limits_{i = 1}^{m} \mathop \sum \limits_{j = 1}^{3} y_{i,j} {\text{log}}p_{i,j}^{role} = - \frac{1}{M}\mathop \sum \limits_{i = 1}^{m} {\text{log}}p_{i}^{role} } \\ \end{array}$$

The loss function for role attribute recognition is defined as the negative log-likelihood of the multi-classification tasks. Where y is the ground truth and (*i*, *j*) denotes the* j*-th label component of the *i*-th relation. $p_{i,j}^{role}$ denotes the probability of being predicted as the *j*-th role under the *i*-th relation. Since only one value of the *j* component in $y_{i,j}$ is 1, the loss function can be expressed as the sum of the corresponding probabilities of M relation truth labels. And since we use a masking mechanism for the FC fully connected layer, i.e., an all-zero mask for FC inputs with no relations, $L_{role}$ is actually the sum of the losses of finite relations.

#### Training detail

From Eq. (1), it is clear that the triple prediction requires the participation of two independent entities. Therefore, we add an additional double-weight (α = 2) for $L_{entity}$. Finally, the total loss is calculated as the sum of entity extraction loss, relation extraction loss, and triple extraction loss.

18 $$\begin{array}{*{20}c} {L_{total} = \alpha L_{entity} + L_{relation} + L_{role} } \\ \end{array}$$

We minimize $L_{total}$ and train the model in a two-step method. First, the entity extractor and the relation extractor are trained synchronously so that the model has the best initialization parameters, and then the whole model is jointly trained in an end-to-end manner.

### Experiments

#### Datasets

To facilitate the comparison of the triple extraction model based on role attribute recognition with other popular approaches, we evaluate our model on two public datasets NYT and WebNLG. There are two versions of these two datasets according to the annotation standard. For the fairness of the experiment, we used the most popular version from the previous work^30^. That is, we selected the datasets that are annotated with only the last word of entity. The statistics of the datasets are described in Table 2. We further described the overlapping pattern and the number of triplets per sentence in the test set.

**Table 2:** Statistics of datasets used in our experiments where N is the number of triples in a sentence.

Dataset	Train	Valid	Test	Details of Test Set			Number of triplets			Relations
				Normal	SEO	EPO	N = 1	N > 1	Triples	
NYT	56195	5000	5000	3266	1297	978	3244	1756	8110	24
WebNLG	5019	500	703	245	457	26	266	437	1591	171

#### Evaluation

We use partial matching as an evaluation metric for comprehensive experiments, i.e., an extracted triple is regarded as correct only if it is an exact match with ground truth, which means the last word of entities of both subject and object and the relation are all correct. Meanwhile, we report the standard Precision (Prec.), Recall (Rec.), and F1-score as in line with all the baselines.

#### Implementation details

Our model is implemented based on Tensorflow and Keras. Our BERT encoder uses the BERT-Base-Cased version, which contains 12 transformer layers and the last hidden layer output size is 768 dimensions. And the single-layer BiLSTM encoder also outputs the 768-dimensional vector. We set the maximum length of sentences to 100 and the batch size on both NYT and WebNLG datasets to 10. During model training, the learning rate of the neural network is set to 1e-5, and the Adam optimizer is used for adaptive adjustment of the weight parameters. The thresholds for both entity and relation recognition are set to 0.5. We trained the model on two RTX2080Ti graphics cards with 11G video memory. We use an early stopping strategy to prevent the model from overfitting, i.e., training is stopped if the performance on the validation set does not improve within 10 epochs.

#### Baseline methods

We compare our model with the following baselines: (1) GraphRel^32^; (2) ETL-Span^33^; (3) WDec^19^; (4) CasRel^15^; (5) TPLinker^1^; (6) CGT^34^; (7) PRGC^30^; (8) LAPREL^22^; (9) RGAM^35^; (10) BiRTE^36^. All the reported results of the baseline models are directly taken from the original literature. We refer to our model as RoleAttrTE (Triple Extraction based on Role Attributes).

### Experimental results and analysis

#### Main results

The main results are shown in Table 3. Compared with other baselines on WebNLG, our proposed model achieves the same level of best results in terms of F1, and achieves almost all the best results in terms of accuracy. RoleAttrTE's F1 results at NYT were slightly worse, but still competitive. This is consistent with the recognition that the error propagation problem of the pipeline method is detrimental to accuracy, but RoleAttrTE brings more benefits in eliminating redundant predictions and facilitating frame construction. On both datasets, RoleAttrTE outperforms most methods such as the table-filling model TPLinker, and the cascade decoding model CasRel, which proves the validity of our hypothesis. This is very meaningful because it shows that: first, the method of low-level feature separation and high-level concept fusion is effective; second, entity role attributes can be identified as key labels for triple extraction.

**Table 3:** Main results, where the bolded marks are the highest scores, and the baseline result data comes from the original literature.

Model	NYT			WebNLG		
	Prec	Rec	F1	Prec	Rec	F1
GraphRel	63.9	60.0	61.9	44.7	41.1	42.9
ETL-Span	85.5	71.7	78.0	84.3	82.0	83.1
WDec	94.5	76.2	84.4	-	-	-
CasRel	89.7	89.5	89.6	93.4	90.1	91.8
TPLinker	91.3	92.5	91.9	91.8	92.0	91.9
CGT	**94.7**	84.2	89.1	92.9	75.6	83.4
PRGC	93.3	91.9	92.6	94.0	92.1	93.0
LAPREL	90.7	91.4	91.1	91.7	91.5	91.6
RGAM	90.6	92	91.3	93.5	91.9	92.6
BiRTE	92.2	**93.8**	**93.0**	93.2	**94.0**	**93.6**
RoleAttrTE	91.8	91.1	91.4	**94.8**	92.4	**93.6**

#### Analysis on submodule

RoleAttrTE is essentially a two-stage model. The entity and relation extraction in the first stage will naturally affect the triple extraction in the second stage, so it is necessary to analyze the performance of these two submodules first. Table 4 gives the results of RoleAttrTE for extracting entities and relations on the two datasets. In terms of relation extraction, the model has similar F1 results on both datasets with a gap of only 0.3%, and the gap in recall is only 0.8% in the case of close precision. This indicates that the relation extraction submodule based on the attention mechanism works stably and performs well. In terms of entity extraction, RoleAttrTE achieves an impressive F1 value of 98.4% on WebNLG, while the result on NYT lags significantly by 3.1%. The reason for this is that the large size of the NYT dataset and the large number of entity types make the entity extraction submodule appear to have insufficient learning ability. This weakness will be improved when a larger parameter version of BERT is considered. Combined with the results in Table 3, we can learn that an important reason for the poor performance of RoleAttrTE on NYT is the poor entity extraction performance.

**Table 4:** Performance of our model for two tasks of entity extraction and relation extraction on two datasets.

Task	NYT			WebNLG		
	Prec	Rec	F1	Prec	Rec	F1
Entity	94.4	96.3	95.3	98.2	98.6	98.4
Relation	95.9	96.0	95.9	95.7	96.8	96.2

For the triple extraction submodule, we conduct a detailed set of evaluations on RoleAttrTE, which includes six cases. ① RoleAttrTEadd: additive feature fusion method. That is, the concatenate operation (;) in Eq. (13) is replaced by the addition operation (+). ② RoleAttrTE2w: dual-entity feature extraction method. That is, the two W_e_ in Eq. (13) are set to W_s_ and W_o_ for extracting the features of subject and object, respectively. ③ RoleAttrTEncls: no global contextual attention feature fusion method. That is, the calculation of Eq. (14) is not performed, while Eq. (15) becomes h_f_=h_t_. ④ RoleAttrTEwhole: single network prediction method. That is, instead of using a relation-specific classification network for entity role attribute recognition, only a fully connected network is used for all relation types with prediction labels {RF, RB, Na}. ⑤ RoleAttrTEwholeM: the same as case 4 uses a single fully connected network for prediction, but the prediction labels are { R_1_F, R_1_B, …, R_m_F, R_m_B, Na}, where m represents the relation type. ⑥ RoleAttrTE: The method proposed in “Methods” section. The results in Table 5 show that our proposed method has the best performance for the following reasons. ① The additive feature fusion method may cover part of the original valid semantic information of entities and relations. ② The dual-entity feature extraction method specifies the order of entity role attributes in advance, which is not good for predicting reverse RB type labels. ③ Obviously, CLS global semantics must contain valuable information for triple extraction. ④ It is difficult for a single prediction network to distinguish the patterns of each relationship itself, and a single prediction label cannot be used to simply replace the entity role attributes of each relationship.

**Table 5:** Evaluation of comparison methods for feature fusion and prediction of triple extraction submodule.

Task	NYT			WebNLG		
	Prec	Rec	F1	Prec	Rec	F1
RoleAttrTE_add_	90.2	90.1	90.1	93.1	91.4	92.2
RoleAttrTE_2w_	90.9	90.4	90.6	93.9	91.5	92.6
RoleAttrTE_ncls_	90.8	90.6	90.7	92.7	91.8	92.2
RoleAttrTE_whole_	51.2	50.0	50.6	36.3	20.1	25.8
RoleAttrTE_wholeM_	80.6	79.5	80.0	70.2	68.5	69.3
RoleAttrTE	**91.8**	**91.1**	**91.4**	**94.8**	**92.4**	**93.6**

#### Analysis on different sentence types

To verify the ability of RoleAttrTE in handling sentences with overlapping or multiple relations, we divided the two data sets according to the overlapping type and the number of triples contained in the sentences. The results of comparison with the typical baseline model are shown in Table 6. It is observed that RoleAttrTE outperforms GraphRel, CasRel, TPLinker and LAPREL in almost all tested items, and has similar performance to PRGC and BiRTE. The extraction effect of RoleAttrTE is stable and does not fluctuate greatly with the increase of sentence complexity. The results show that RoleAttrTE has advantages in dealing with overlapping problems.

**Table 6:** F1 scores on sentences with different overlapping types and different numbers of triples.

Model	NYT								WebNLG							
	Normal	EPO	SEO	N = 1	N = 2	N = 3	N = 4	N ≥ 5	Normal	EPO	SEO	N = 1	N = 2	N = 3	N = 4	N ≥ 5
GraphRel	69.6	51.2	58.2	71.0	61.5	57.4	55.1	41.1	65.8	38.3	40.6	66.0	48.3	37.0	32.1	32.1
CasRel	87.3	92.0	91.4	88.2	90.3	91.9	94.2	83.7	89.4	94.7	92.2	89.3	90.8	94.2	92.4	90.9
TPLinker	90.1	94.0	93.4	90.0	92.8	93.1	**96.1**	90.0	87.9	95.3	92.5	88.0	90.1	94.6	93.3	91.6
LAPREL	89.0	93.1	92.6	89.0	92.5	93.3	95.2	86.8	87.0	97.1	92.5	87.2	91.2	94.6	92.9	90.2
PRGC	91.0	**94.5**	94.0	91.1	93.0	93.5	95.5	**93.0**	90.4	**95.9**	93.6	89.9	91.6	95.0	94.8	**92.8**
BiRTE	**91.4**	94.2	**94.7**	**91.5**	**93.7**	**93.9**	95.8	92.1	90.1	94.3	**95.9**	90.2	**92.9**	**95.7**	94.6	92.0
RoleAttrTE	89.6	93.6	93.1	89.9	91.5	93.0	95.9	89.9	**93.9**	94.7	94.6	**90.8**	91.8	95.2	**94.9**	92.6

#### Analysis on prediction errors

**Figure 2:**
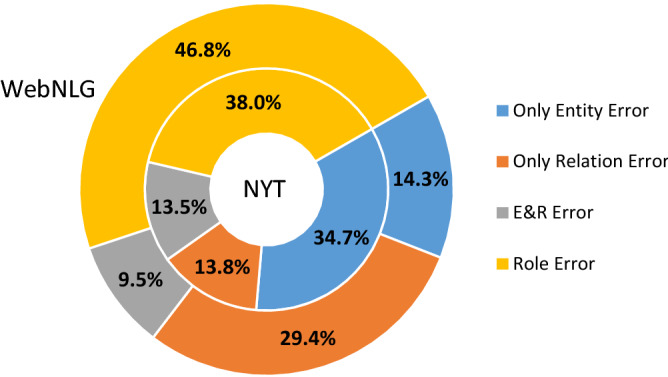
Comparison of triple prediction error categories.

We classify the triples of extraction errors into the following four categories. Case 1: The triple contains only incorrect entity (at least one). Case 2: The triple contains only incorrect relation. Case 3: Cases 1 and 2 occur simultaneously. That is, both the entity and the relation contained in the triplet are incorrect. Case 4: The extraction of both entities and relations is correct, but the prediction of entity role attributes is incorrect (including misclassification as NA). The following two observations can be obtained from Fig. 2. Observation 1: The total sum of errors caused by the first stage of the model (cases 1, 2, and 3, all areas except yellow in Fig. 2) is 62% and 53.2%, respectively, both exceeding the percentage of errors in the second stage (case 4, yellow area in Fig. 2). Observation 2: When only the first stage is observed, the highest percentage of entity errors is found on NYT (cases 1, 3), while the higher percentage of relation errors is found on WebNLG (cases 2, 3), which is fully consistent with the data performance in Table 4. Thus, the following conclusions are drawn. First, it can be seen from observation 1 that the overall performance of the model can be improved by improving the accuracy of entity or relation extraction. Clearly, it is much easier to improve the two subtasks independently than to construct methods that fuse the features associated with both tasks. Therefore, there is still much room for progress in traditional pipeline methods. Second, it can be seen from observation 2 that relation features play a significantly more important role in the approach of this paper. An increase in the error rate of the previous relation classification leads to a decrease in the accuracy of the later role attribute recognition. We believe that the insufficient supply of relation information in the triple feature fusion process is the main reason for this phenomenon, and a better introduction of relation correlation features can improve the prediction performance of the model.

## Conclusions

This paper proposes a simple, understandable and effective ERE joint extraction model based on role attribute recognition of entities. The framework model adopts the strategy of low-level feature separation and high-level concept fusion, which naturally simulates the cognitive process of human triple extraction, and thus has strong interpretability. Meanwhile, the model can effectively solve the redundant prediction and overlapping relation problems caused by the coupling of entity and relationship features. The evaluation of the model from different aspects proves the correctness of our conjecture. ① The entity role attributes are salient features of the triple and can be identified from the holistic triple fusion features. ② In addition to their own meanings, relation features contain features of how the elements of the triple match, and can play a role in correctness discrimination in the triple fusion features. To the best of our knowledge, this paper is the first model that uses complete triple and entity role attributes as recognition objects. Experimental results show that our model achieves the same technical level as the latest SOAT on two benchmark datasets. Further analysis also demonstrated the outstanding performance of the model in handling sentences with overlapping and multiple relations.

## Data Availability

Our dataset access is open. Details of our dataset can be found online at https://gitee.com/JingXatu/RoleAttrTE/tree/master/Datasets.

## Code availability

The model source code is available at: https://gitee.com/JingXatu/RoleAttrTE.
